# WISIT vaccines based on IL-31-derived peptides as a novel therapeutic approach for chronic pruritic dermatoses

**DOI:** 10.1371/journal.pone.0318293

**Published:** 2025-02-11

**Authors:** Sabine Schmidhuber, James Dickie, Mihály Cserepes, József Tóvári, Achim Schneeberger, Markus Mandler

**Affiliations:** 1 Tridem Bioscience GmbH & CoKG, Campus Vienna Biocenter, Vienna, Austria; 2 MODUS Research and Innovation, Unit D Tayside Software Centre, Gemini Crescent, Dundee Technology Park, Dundee, United Kingdom; 3 KINETO Lab Ltd., Budapest, Hungary; Anhui Polytechnic University, CHINA

## Abstract

Vaccines are a promising therapy for the treatment of chronic conditions such as pruritus. IL-31 has been identified as an important mediator of itch. By targeting IL-31 signaling with immunotherapy, CP can be effectively alleviated. However, self-antigens such as IL-31 are highly tolerated, which has rendered conventional conjugate vaccines (CCVs) ineffective at generating sufficient antibody (Ab) responses to alleviate CP. Novel Win the Skin Immune System Trick (WISIT) vaccines however have been shown to induce substantially stronger Ab responses than CCVs in Parkinson’s Disease, and so may be capable of overcoming IL-31 tolerance to effectively treat CP. In this report, WISIT vaccines presenting ten different IL-31-specific peptides were compared to CCVs presenting the same peptides. Multiple response parameters were assessed, including Ab titers induced, avidity of these Abs, and IL-31 signaling inhibition. Results demonstrated that WISIT vaccines outperform CCVs across all investigated metrics, culminating in the identification of 3 promising candidate WISIT vaccines to be taken forward for further clinical development. This report thus provides evidence that the improved immunogenicity of WISIT vaccines is not disease-specific and that WISIT vaccines may also be translated to treat dermatological disorders. Further preclinical development will be necessary to prepare the identified IL-31 targeting WISIT vaccine candidates for clinical testing.

## Introduction

Chronic pruritus (CP) is a common symptom associated with a vast array of dermatologic, neurologic, psychogenic/psychosomatic, and systemic diseases [1–3]. Lifetime prevalence of CP in the general population is around 25% [4], often placing a high burden on patient quality of life and the economy [5–8]. CP is often difficult to treat, and whilst mild and local cases may be treated topically, severe cases usually rely on systemic treatments that offer varying degrees of success and economic viability. More common pruritic dermatologic conditions include Atopic Dermatitis, Psoriasis and Prurigo Nodularis (PN) [9]. The latter is an intensely pruritic skin disease with characteristic hyperkeratotic nodules on the trunk and extremities. Despite its tremendous disease burden, PN is largely understudied, and available therapeutics are limited.

The exact underlying mechanisms of CP are not yet fully understood, but recent preclinical and clinical studies strongly implicate type 2 skin inflammation-associated interleukins (ILs).

IL-4 and IL-13 play major critical roles in the inflammatory processes [10], and the current most advanced treatment for PN (Dupilumab) targets IL-4 and IL-13 signaling [11,12]. However, IL-31 appears to be pivotal to the itching component of CP [10,13], and so attention in CP treatment has recently turned towards IL-31 [14–21]. IL-31 is a Th2-derived cytokine related to the IL-6 subgroup, composed of four helices named A–D from the N terminus to the C terminus [22]. The receptor for IL-31 is a heterodimeric complex composed of IL-31 receptor A (IL-31RA) and the oncostatin M receptor (OSMR), which when bound to IL-31 activates cell signaling pathways including JAK/STAT, PI3K/AKT, and MAPK. IL-31 orchestrates the process of pruritus through dual pathways, through stimulation of inflammatory cells and through neuronal sensitization [23].

To date, only a single IL-31 signal-targeting agent has passed late-stage clinical trials for PN treatment. Nemolizumab is an anti-IL-31RA monoclonal antibody (mAb) that recently passed stage 3 clinical trials, that significantly reduced the symptoms of PN and cleared the skin in a third of patients treated [24]. Vixarelimab is the only other IL-31 signal-targeting agent currently in late-stage clinical development for PN, which instead targets OSMR. Vixarelimab recently passed stage 2 clinical trials, reducing symptoms and clearing the skin in a third of PN patients, offering comparable efficacy to Nemolizumab [25].

Whilst Nemolizumab and Vixarelimab both target different parts of the IL-31 receptor, there are no agents that directly target the IL-31 protein currently under clinical development. The most advanced IL-31 protein targeting therapy was BMS-981164, an anti-IL-31 mAb developed by Bristol-Myers Squibb. However, BMS-981164 was discontinued following the early termination of phase I clinical trials in 2015, and results on the clinical performance of BMS-981164 were never published [12]. The only other anti-IL-31 therapy in development is Lokivetmab, a caninized anti-IL-31 mAb approved for treating canine dermatitis, that is not suitable for human use [26].

Notably, all the above-described anti-pruritic therapies (Nemolizumab, Vixarelimab, Dupilumab, BMS-981164 and Lokivetmab) are passive therapies, in which mAbs are infused into recipients. Whilst passive therapies offer advantages in terms of control and specificity, they also suffer from high costs, adverse side effects, lack of durability and need for frequent administration. For treating chronic and frequently reoccurring conditions such as CP, these barriers are critically limiting to their widespread clinical uptake and use. Vaccines on the other hand are generally very safe and economical to produce and administer and would be far better suited for the treatment of CP than mAbs. However, the greatest limitation of vaccines currently is their efficacy, which is very poor when targeting tolerated self-antigens such as cytokines and their receptors. To achieve clinical benefits, therapeutic Abs must meet a certain minimum immunogenic threshold of titer and avidity. Therapeutic mAbs can be artificially manipulated to meet this immunogenicity threshold, but current conventional vaccines are unable to induce a sufficient Ab response in vivo. As a result, there are no vaccines currently under clinical development for CP, although vaccines based on virus-like particles are currently under development for use in canines [27] and horses [28].

However, Tridem Biosciences recently developed a novel vaccine platform termed WISIT (Win the Skin Immune System Trick), which can produce substantially stronger Ab responses than conventional conjugate vaccines (CCVs). CCVs are composed of a B cell peptide linked to a carrier protein delivering T cell help, such as KLH or CRM197, which is then adsorbed to adjuvants such as Alhydrogel^®^ or MF59. These additional adjuvants are essential for CCVs to attract antigen-presenting cells (APCs) and retain vaccine constructs at the injection site because the subcutaneous or muscle tissues to which CCVs are traditionally delivered are sparsely populated by APCs.

WISIT vaccines on the other hand are small particles that are specifically designed to target and activate APCs. WISIT vaccines incorporate sugar moieties that facilitate uptake into APCs by receptor-mediated endocytosis and subsequent cell activation directed to the chosen target. This novel “Intrinsic Ab Therapy” approach can produce extraordinarily potent Ab responses against the target, based on titer and avidity, when applied intradermally, where dendritic cells (DCs) are abundant, making conventional adjuvants unnecessary [29].

**Fig 1:**
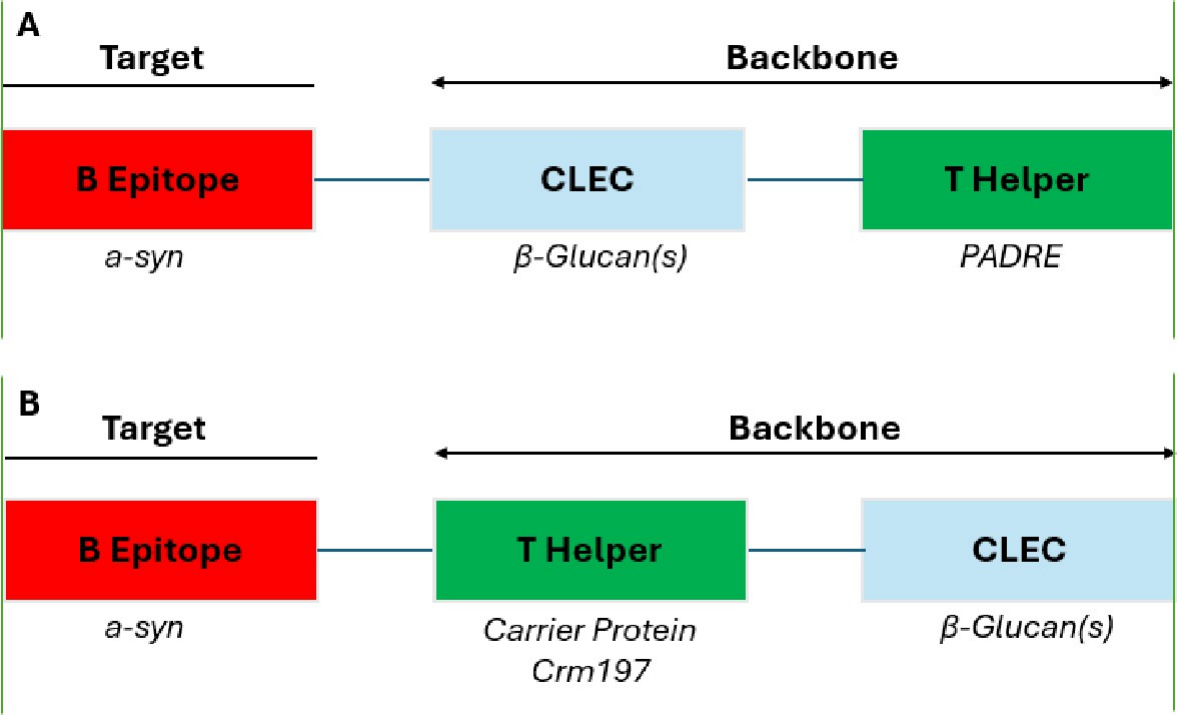
Comparison of A and B-WISIT vaccine structures. Format A: A linear ß-D-glucan/CLEC is central. T helper and B cell peptides are linked to it. PADRE, a synthetic linear pan HLA T helper epitope with proven activity in humans, is used as the T helper element. Format B: The central position is taken by the T helper protein (CRM197), and B cell peptides and ß-D-glucan are linked to it.

There are currently 2 potential formats for WISIT vaccines: Format A (A-WISIT) and Format B (B-WISIT). A-WISIT vaccines are formed of 3 elements: 1) The peptide targets suited to Ab response induction, 2) PADRE, universal pan-helper T cell epitope and 3) β-glucan, a C-type lectin (CLEC) sugar residue. The target peptides and PADRE are physically linked to the β-glucan, which forms the core of A-WISIT. B-WISIT vaccines on the other hand use a conventional carrier (CRM197) as the core, like CVVs, upon which the target peptides and β-glucan are linked (Fig 1).

A-WISIT vaccines are simpler and cheaper to fabricate than B-WISIT vaccines and have already been successfully demonstrated in the context of Parkinson’s Disease, targeting aSyn to provide disease-modifying effects [29]. These previous results demonstrated that A-WISIT vaccines were superior to CCVs in all aspects of the elicitation of target-specific Abs [29]. In brief, though the same aSyn antigens were used in both vaccine types, the levels of A-WISIT vaccine-induced Abs exceeded the Ab levels obtained with CCV by a factor >10, and the affinity of A-WISIT vaccine-induced Abs to the target antigen also exceeded that of CCV by a factor of >10. Furthermore, the dose-response relationship for A-WISIT was uniquely linear (up to 100μg tested) as opposed to the plateau-type (5–100μg) relationship of CCV.

B-WISIT vaccines offer different benefits, due to their use of CRM197 as the backbone. As an external antigen, CRM197 minimized the risk of T cell autoimmunity and may produce even stronger Ab responses than A-WISIT vaccines. Furthermore, CRM197 is already widely utilized in many clinical products, such as Menveo or different Prevnar variants. Hence, B-WISIT technology would be relatively easy for existing CCV developers to adopt. However, B-WISIT vaccines have not yet been demonstrated in disease.

Following the success of A-WISIT vaccines targeting aSyn, it stands to reason that the WISIT concept, targeting peptide-based conjugate vaccines to APCs via β-glucan receptors, could also be successfully deployed to target IL-31 via Abs for the treatment of CP, and expanded to demonstrate B-WISIT vaccines. In this report, evidence is presented for the potential of B-WISIT vaccines to target IL-31, including characterization of vaccine-induced Ab responses such as Ab levels, avidity, and isotype patterns.

## Materials and methods

### Generation of vaccine candidates

All peptides used were synthesized by FMOC solid-phase peptide synthesis (INTAVIS Peptide Services GMBH; Tübingen, Germany). Peptides were coupled to the carrier CRM-197 (EcoCRM^™^-Maleimide, Fina Biosolutions LCC, Rockville, MD, USA) by maleimide–thiol coupling [29]. Briefly, excess peptide containing free sulfhydryl-groups via a C-terminal Cysteine residue was added to EcoCRM^™^-Maleimide for coupling in a 200 mM Na-phosphate buffer (pH 6.8) and subsequently dialyzed with PBS. Ellmann reagent 5,50-dithio-bis-(2-nitrobenzoic acid; Thermo Fisher Scientific, Waltham, MA, USA) was used for quantifying free sulfhydryl groups in solution and assess coupling efficacy.

For subsequent WISIT vaccine production, CLEC/β-glucan was first activated by mild periodate activation (using NaIO4, Thermo Fisher Scientific, Waltham, MA, USA) to open furanose rings of the ß-glucans between vicinal diols leaving aldehyde groups as substrate for the subsequent hydrazone bond formation. Peptide-CRM conjugates were coupled to activated CLEC/β-glucan by hydrazone formation in the presence of heterobifunctional maleimide-and-hydrazide crosslinker (Thermo Fisher Scientific, Waltham, MA, USA) in PBS (pH 7,2) and conjugates were subsequently dialyzed with PBS. The carbohydrate concentration in conjugates was estimated using the anthrone method.

For CCVs, the respective Peptide-CRM conjugate was adjuvanted with Alhydrogel (Invivogen, Toulouse, France). Vaccine doses for both WISIT vaccines and CCVs were based on the amount of conjugated peptides.

### Animal experiments

For all experiments, 8–10-week-old female BALB/c mice (n = 10) were immunized once every two weeks for 4 weeks (3 injections total) with either the WISIT vaccine or CCV. Except where stated otherwise, CCVs were applied subcutaneously (s.c.) with Alhydrogel^®^ adjuvant, and WISIT vaccines were applied intradermally (i.d.). Blood samples were taken one day before each vaccination and two weeks after the last application, unless otherwise indicated. Blood was processed to yield plasma, which was subjected to ELISA to determine the Ab titers against the respective peptides and recombinant human IL-31 (hIL-31) protein (Abcam, Cambridge, UK).

### Animal welfare

All animal procedures were conducted at Kineto Lab Ltd. (Budapest, Hungary) in compliance with national Hungarian legislation. Experiments were performed under approval number PE/EA/448-7/2021 and according to HLASA recommendations for animal use. Injection of the compounds were performed in ketamine/xylazine anesthesia in order to minimize suffering. Body weight was monitored weekly, as well as general appearance and activity of the mice. All animals maintained the score of zero during the experiment. Small blood samples up to 70 μL volume were collected from the retroorbital plexus following temporary anesthesia using brief isoflurane inhalation. All animals were sacrificed at the termination of the experiments by brief isoflurane anesthesia followed by cervical dislocation, and total blood was taken by cardiac puncture. For the welfare of the animals, all experimental groups were kept one cage per group, no animals were kept alone. We provided hiding space using cardboard mesh and tubes. The animal house works with a controlled environment with temperature of 24–25°C, relative humidity of 60%, and 12/12hour light/dark cycle lighting.

### Vaccine-induced antibody titer determination

Standard ELISA technology was used to measure levels of vaccine-induced Abs in plasma. Briefly, ELISA plates (Nunc Maxisorb; Thermo Fisher Scientific, Waltham, MA, USA) were coated with either 1 μg/mL hIL-31 (Abcam, Cambridge, UK) or respective IL-31 derived-peptide-bovine serum albumin (BSA) conjugates (1μM) using 50 mM sodium carbonate buffer overnight at 4°C. Plates were blocked with 1% BSA, and plasma samples were serially diluted in the plates. The detection of target-specific Abs was performed with biotinylated anti-mouse IgG (Southern Biotech, Birmingham, AL, USA) and a subsequent colour reaction using Streptavidin-POD (Roche, Basel, Swiss) and TMB. For IgG subtype-specific analyses, biotinylated anti-mouse IgG was replaced by biotinylated anti-mouse IgG1 and IgG2a second-step reagents (Southern Biotech, Birmingham, AL, USA), respectively. Titers were calculated as EC_50_ values using Prism^®^ 9.3 following non-linear regression analysis (four-parameter logistic fit function). Immunogenicity against hIL-31 protein was assessed by quantifying plasma Ab concentrations using an ELISA-based method with a commercially available hIL-31 Ab (R&D Systems, Minneapolis, MN, USA) as standard.

### Fc-Dectin 1 binding

Fc-Dectin-1 binding was analyzed by ELISA using soluble murine and human Fc-dectin-1a receptors (InvivoGen, San Diego, CA, USA) as previously described [29,30]. Briefly, ELISA plates were coated with a reference CLEC (β-Glucan) and non-conjugated CLEC, WISIT conjugates as well as CCV conjugates were tested in a competitive ELISA (increasing doses of conjugates) to demonstrate dectin binding. The detection of remaining murine/human Fc-Dectin1 binding was performed with HRP donkey anti human IgG (Biolegend, San Diego, CA, USA) and a subsequent colour reaction using TMB.

Binding efficacy was calculated as IC_50_ (half maximal inhibitory concentration) values with Prism^®^ 9.3 (GraphPad Inc, San Diego, CA, USA) by non-linear regression analysis (four-parameter logistic fit function). IC50 = concentration in μg/mL of carbohydrate/WISIT conjugate/CCV required to block 50% of Dectin-1 binding to plate bound CLEC (β-Glucan).

### Avidity analysis

The avidity of vaccine-induced Abs to hIL-31 protein was determined in the presence of the chaotropic agent sodiumthiocyanate (NaSCN) by an adapted ELISA. Briefly, ELISA plates (Nunc Maxisorb; Thermo Fisher Scientific, Waltham, MA, USA) were coated with 1μg/ml hIL-31 (Abcam, Cambridge, UK) using 50 mM sodium carbonate buffer, overnight at room temperature (RT). Plates were blocked with 1% BSA, and constant amounts of plasma samples were added and incubated for 1 h. Following plate washing, 100 μL of a chaotropic agent was added at increasing concentrations (0 to 3 M) for 20 min at RT. Plates were washed again, and residual Ab binding was determined. The detection of target-specific Abs was again performed with biotinylated anti-mouse IgG (Southern Biotech, Birmingham, AL, USA) and a subsequent colour reaction using Streptavidin-POD (Roche, Basel, Swiss) and TMB. Absorbance values in the absence of NaSCN were taken as the total effective binding of the specific Ab (100% binding), and subsequent absorbance values in the presence of increasing concentrations of NaSCN were converted to the corresponding percentage of the total bound Ab. The data were fitted to a plot of (% binding) vs. (log) concentration of NaSCN, and linear regression analysis was used to estimate the avidity index. The avidity index was defined as the concentration necessary to decrease the initial absorbance by 50%.

### IL-31 inhibition assay

To investigate the inhibition of native hIL-31 signaling by WISIT vaccine- or CCV induced Abs, A549 cells (ATCC, Virginia, USA) were cultured in F-12K medium (Life Technologies, Tokyo, Japan) containing 10% fetal bovine serum (FBS) (Moregate Biotech, Bulimba, Australia) and 100 units/mL penicillin- 100 μg/mL streptomycin (Life Technologies) in 24- well plates (Corning Inc., Corning, NY, USA) overnight at 4×10^5^ cells/well. Next, the cells were treated with equal amounts of WISIT vaccine- or CVV-induced Abs or commercially available anti-hIL-31-Abs (R&D Systems, Minneapolis, MN, USA) for 2 hours before adding hIL-31. After incubation for 20 minutes, cells were lysed and phosphorylation of STAT3 was analyzed using the PathScan Phospho- Stat3 (Tyr705) Sandwich ELISA Kit (Cell Signaling Technologies, Danvers, MA, USA) at 450 nm.

### Statistical analysis

All statistical analyses were conducted using Prism^®^ 9.3. Data are presented as mean ± standard error of the mean (SEM) and were analyzed with one-way analysis of variance (ANOVA) with post-hoc Tukey’s multiple comparison test unless indicated otherwise. A value of p < 0.05 was considered statistically significant. *p*-values are shown as: *p ≤ .05, **p ≤ .01, ***p ≤ .001, ****P ≤ .0001.

## Results

### Vaccine candidates tested

Ten peptides (7-21aa in length) covering the hIL-31 molecule were selected as potential targets for the developed WISIT vaccines, including 3 peptides from Helix A; 1 from Helix B; 1 from Helix C (both summarized as Helix B/C), and 5 from Helix D (summarized as Helix D and C-terminus; Table 1).

**Table 1:** IL-31-derived peptides used in WISIT vaccines and CCVs: Size and position within the IL-31 molecule.

Name	B-Cell Epitope Length	IL-31 Region
Pep1	14-mer	aa35-70; Helix A
Pep2	9-mer	aa35-70; Helix A
Pep3	7-mer	aa35-70; Helix A
Pep4	15-mer	aa84-119; Helix B/C
Pep5	21-mer	aa84-119; Helix B/C
Pep6	10-mer	aa131-164; Helix D and C-terminus
Pep7	10-mer	aa131-164; Helix D and C-terminus
Pep8	13-mer	aa131-164; Helix D and C-terminus
Pep9	13-mer	aa131-164; Helix D and C-terminus
Pep10	13-mer	aa131-164; Helix D and C-terminus

For B-WISIT vaccines, peptides were conjugated to CRM197 and CLEC/β-glucan. For CCV, peptide/CRM197 conjugates were admixed with Alhydrogel^®^.

### WISIT vaccines targeting IL-31 have a high binding capacity towards antigen-presenting cells

**Fig 2:**
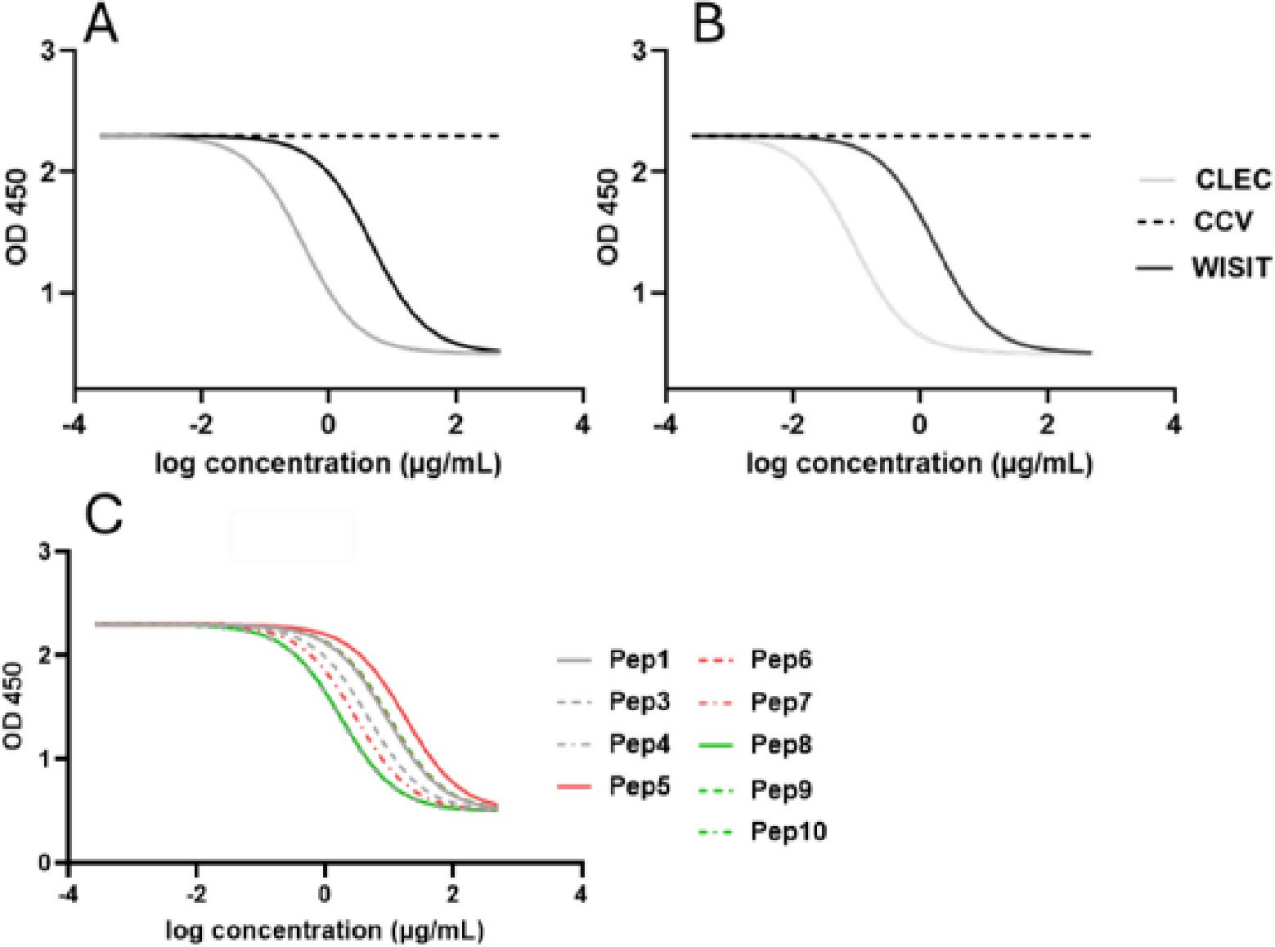
Binding of representative WISIT vaccines and CCVs targeting hIL-31. Comparative analysis of Pep2-based vaccines to murine (a) and human (b) Dectin-1, showing absence of binding by CCVs, and high binding by CLEC/β-glucan and WISIT vaccines. c) Binding of all other WISIT vaccine candidates targeting IL-31 peptides to murine Dectin-1.

Firstly, to determine the biological activity of vaccine candidates, the binding capacity of WISIT vaccines to murine and human Dectin-1 was assessed and compared to CCVs using a competitive ELISA. Dectin-1 is a CLEC/β-glucan pattern recognition receptor (PRR) expressed on various APCs including macrophages, neutrophils and DCs [31]. The comparison showed that CCVs do not interact with either human or mouse Dectin-1, whilst activated CLEC/β-glucan and WISIT vaccine display a high binding capacity to both receptors in vitro (Fig 2).

### WISIT vaccines induce high levels of IL-31 peptide-specific antibodies

**Fig 3:**
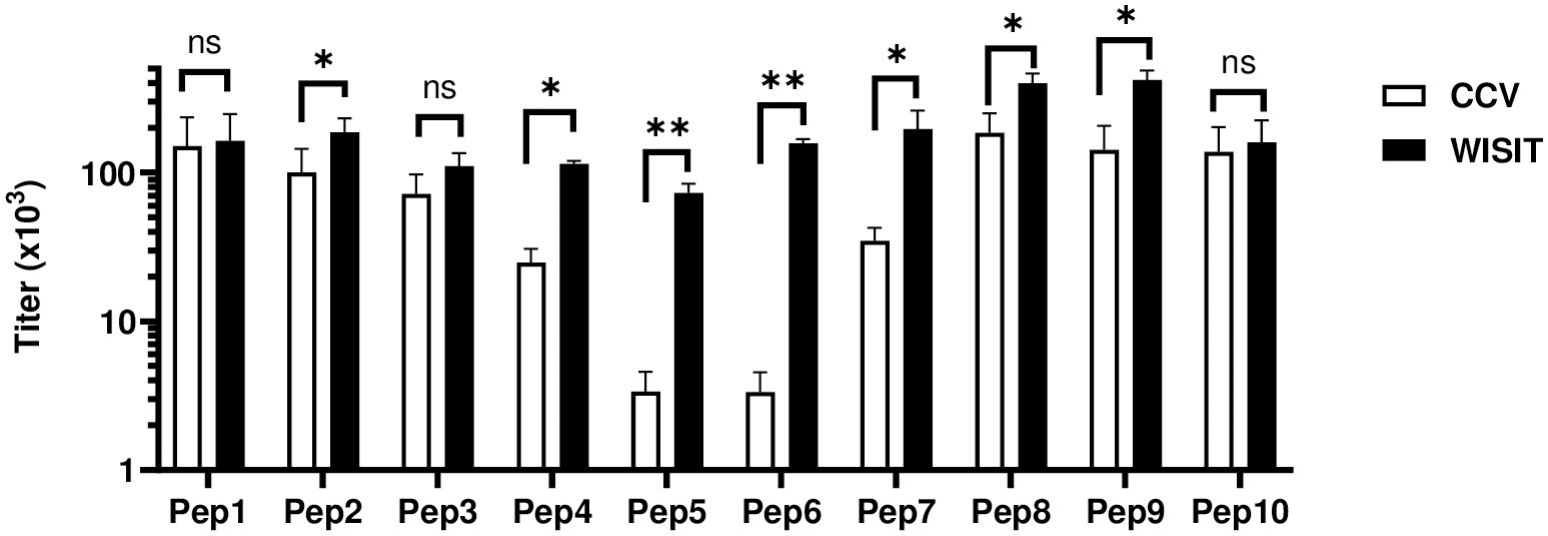
WISIT vaccines induce higher Ab titres than CCVs against 10 peptides covering the IL-31 molecule in Balb/c mice (n = 10/group). Bars and error bars indicate the mean values of Ab levels + SEM. Statistical differences were evaluated by one-way ANOVA and Tukey’s multiple comparison test. * p < 0.05; ** p < 0.01; ns not significant.

To demonstrate the immunogenicity of WISIT vaccines, mice were injected repeatedly with either WISIT vaccines or CCVs presenting one of the ten IL-31 peptides (Table 1), and the resulting Ab levels induced were assessed by respective peptide ELISAs. Results showed that WISIT vaccines induced higher Ab titers against hIL-31-derived peptides than CCVs for all peptides screened (Fig 3). Of note, peptides Pep5 and Pep6 induced relatively low Ab titers when introduced by CCV format but produced robust Ab titers when introduced by WISIT vaccines.

### WISIT vaccines induce high levels of IL-31 protein-specific antibodies

**Fig 4:**
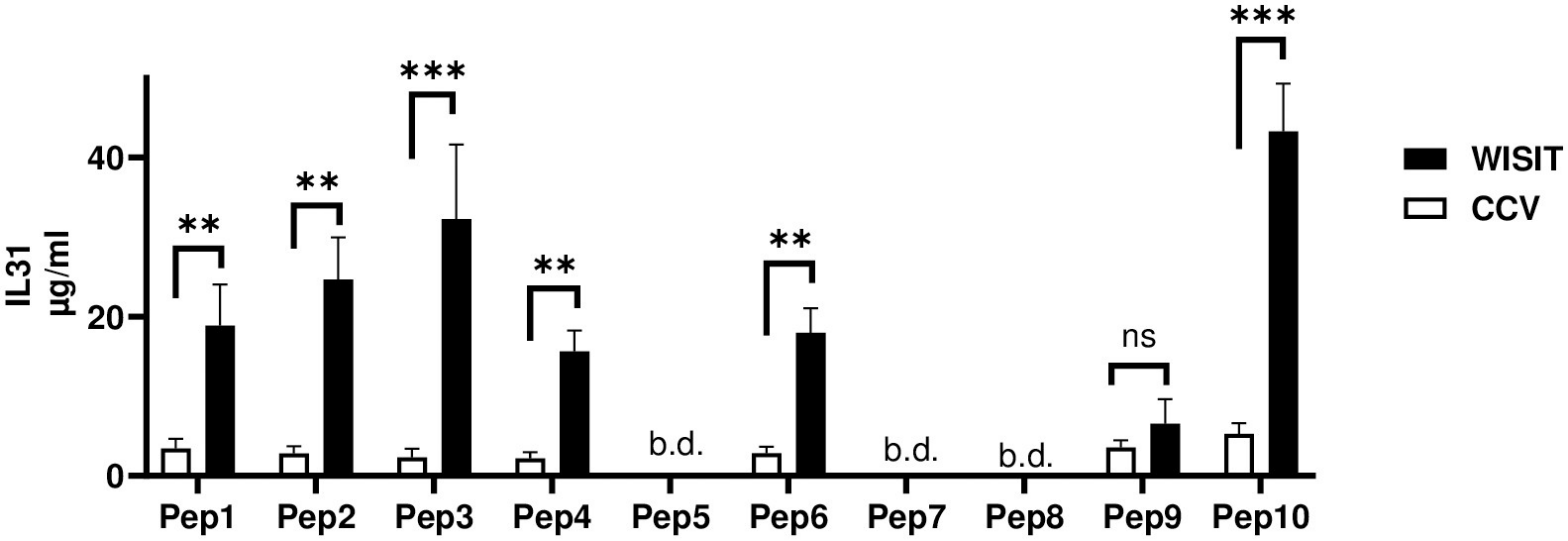
WISIT vaccines targeting IL-31 peptides induce higher Ab titres than CCVs against IL-31 protein in Balb/c mice (n = 10/group). Bars and error bars indicate the mean values of Ab levels + SEM. B.d. = below detection; Statistical differences were evaluated by one-way ANOVA and Tukey’s multiple comparison test. * p < 0.05; ** p < 0.01; ***p<0.001; b.d. below detection limit; ns not significant.

Following the demonstration that WISIT vaccines produced higher yields of Abs specific to IL-31 peptides, the ability of these Abs to target the full-length hIL-31 protein was then assessed by ELISA using commercial anti-hIL-31 Ab as a standard. Results showed that the vaccines targeting peptides Pep5, Pep7 or Pep8 did not induce any detectable Ab response to hIL-31 protein for either WISIT vaccines or CCVs, suggesting a lack of Ab cross-reactivity towards full-length hIL-31 protein for vaccines based on these peptides. For all seven remaining peptides, WISIT vaccines induced higher levels of Abs targeting hIL-31 protein (Fig 4).

### Intradermal application is optimal for WISIT vaccines targeting IL-31

**Fig 5:**
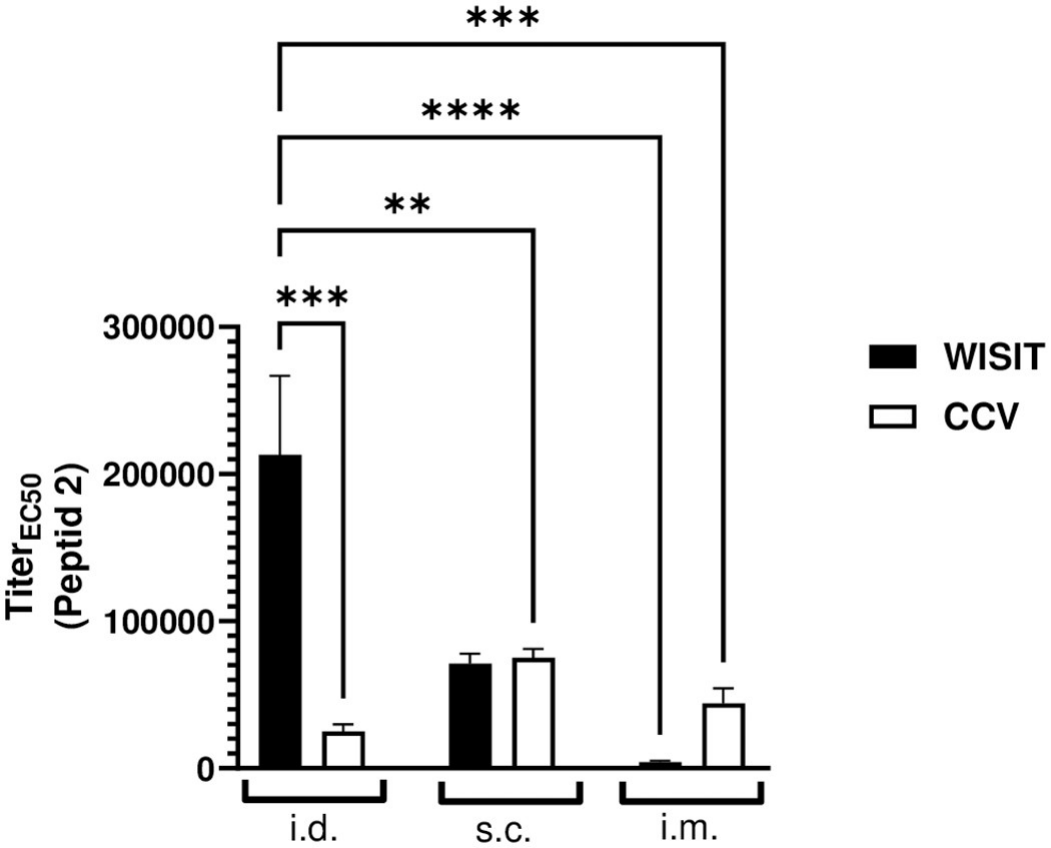
Intradermal administration is the optimal application route for WISIT vaccines targeting Pep2 in Balb/c mice (n = 6/group), comparing intradermal (i.d.), subcutaneous (s.c.) and intramuscular (i.m.) routes. CCV i.d. animals received peptide/CRM conjugates without Alhydrogel^®^. Bars and error bars indicate the mean + SEM. Statistical differences were evaluated by one-way ANOVA and Tukey’s multiple comparison test. ** p < 0.01; ***p<0.001; ****p<0.0001.

To compare vaccine application routes, one of the WISIT vaccine candidates (Pep2) and the corresponding CCV were administered by either subcutaneous, intramuscular, or intradermal routes, and the resultant Ab titers generated were measured. Results demonstrated that when applied subcutaneously, the WISIT vaccines produced only comparable results to CCV, and when applied intramuscularly, WISIT achieved minimal Ab response. When applied intradermally, however, the WISIT vaccines generated between 3- to 8-fold higher Ab titers than any other setting tested (Fig 5). This could be attributed to the high amount of DCs present in the dermis. Similar results have been seen previously for A-WISIT vaccines targeting aSyn relying on peptide-β-glucan conjugates [29].

### WISIT vaccine-induced antibodies have higher avidity to their target antigen

Whilst Figs 3 and 4 demonstrate that WISIT vaccines induce higher Ab levels to target peptides and proteins than CCVs, immunogenicity is a combined metric dependent on both Ab concentration and target avidity [32].

**Fig 6:**
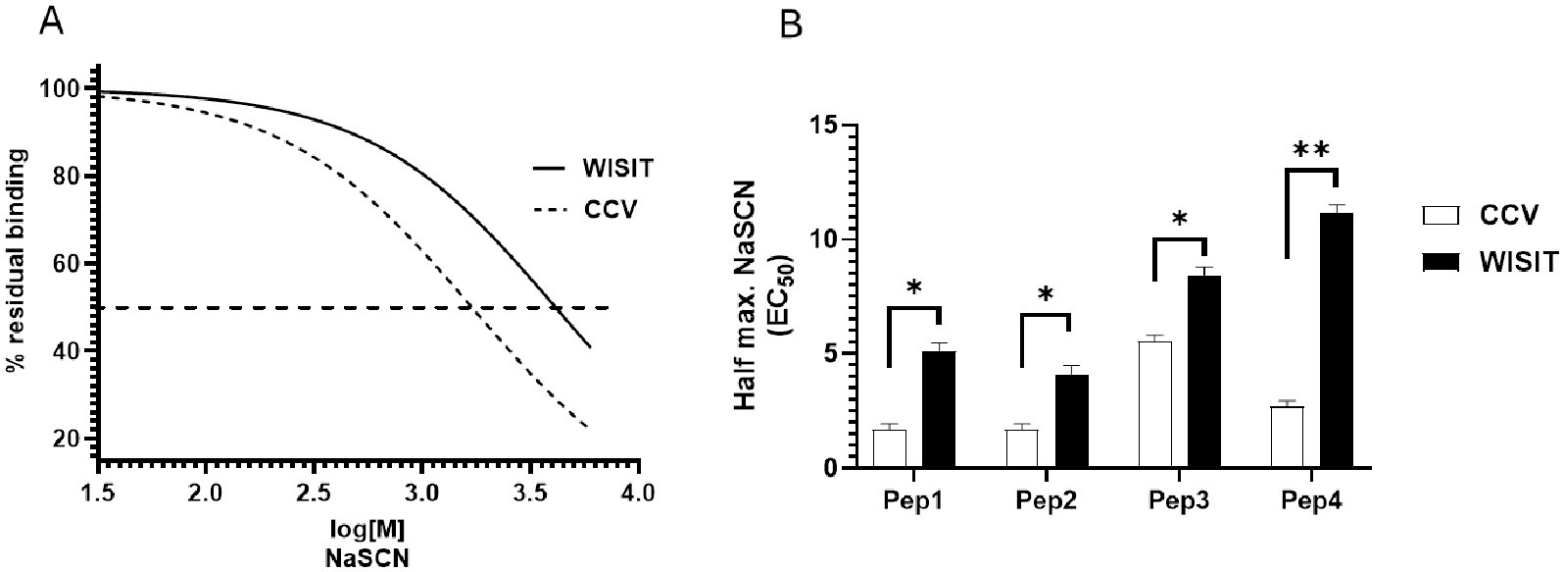
WISIT vaccine-induced Abs have higher avidity for their cognate antigen than CCV-induced Abs in Balb/c mice. (A) Representative graph for residual binding towards hIL-31 for Pep2-WISIT and Pep2-CCV-induced antibodies after challenging with increasing concentrations of chaotropic thiocyanate ions (0.25 M to 6 M) and (B) avidity indices determined. Bars and error bars indicate the mean + SEM of two different experiments performed in triplicate. Statistical differences were evaluated by one-way ANOVA and Tukey’s multiple comparison test. * p < 0.05; ** p < 0.01.

To compare Ab avidity, selected WISIT vaccine candidates and the corresponding CCVs were assessed by NaSCN displacement ELISA. Results showed that the WISIT vaccine-induced Abs demonstrated higher relative avidity compared to CCVs, as the EC_50_ for NaSCN displacement was significantly higher for WISIT vaccine Abs than CCV Abs (Fig 6).

### WISIT vaccines induce more balanced IgG subtypes

**Fig 7:**
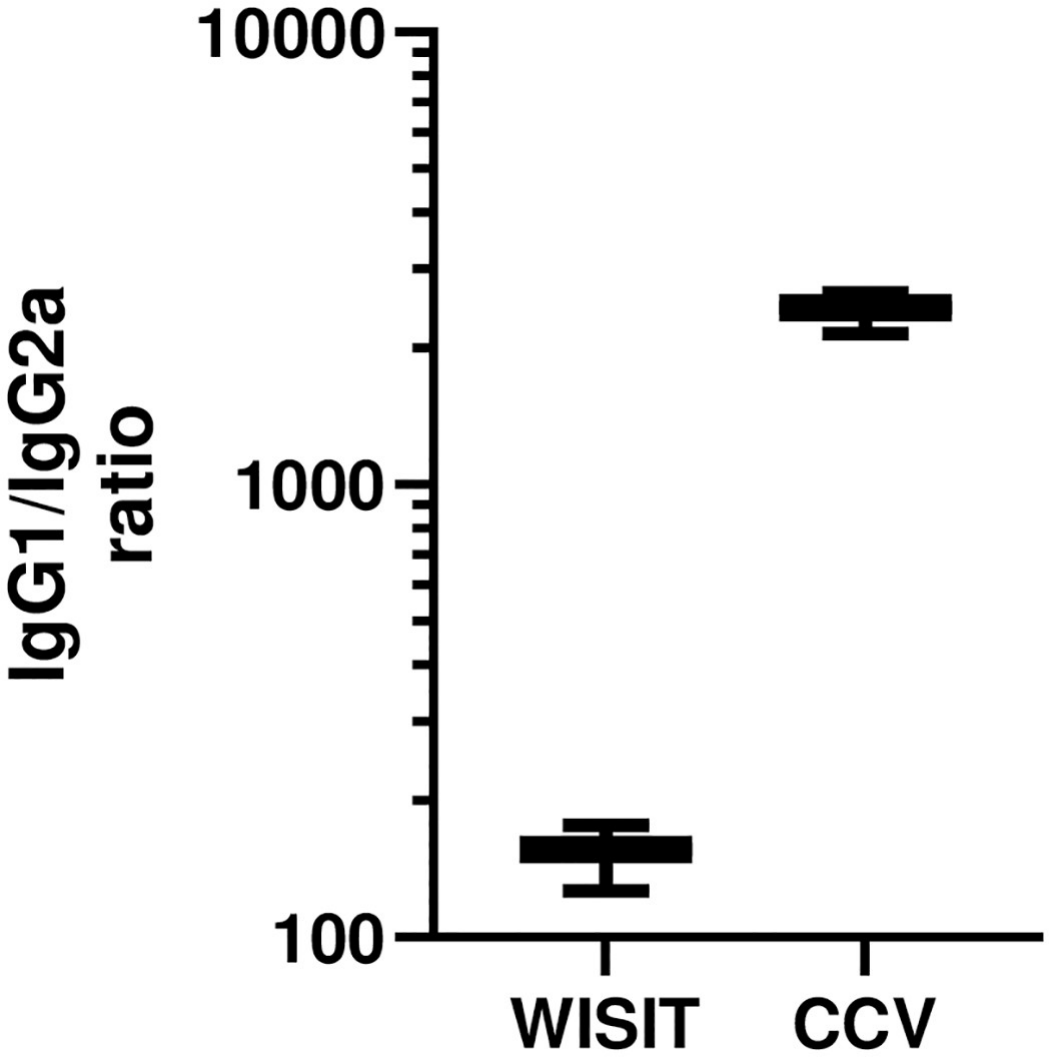
WISIT vaccines induce a more balanced IgG1/IgG2 ratio than CCVs in Balb/C mice. Balb/C mice were immunized with either WISIT- or CCV vaccines and anti-peptide-specific IgG1 and IgG2a responses were determined via ELISA for each peptide (Pep1—Pep10), separately. IgG1/IgG2a ratio represents the ratio between the corresponding ELISA titers (Pep 1–10 were grouped for WISIT and CCV) and is depicted as mean ± SEM (n = 5/group).

In addition to avidity, the Ab response was further characterised by exploring the IgG subtypes induced. In mice, IgG1 production is primarily induced by Th2 cytokines (anti-inflammatory), whilst IgG2 is primarily induced by Th1 cytokines (anti-viral/anti-cancer) [33]. To compare IgG subtypes induced, WISIT vaccine candidate—and the corresponding CCV- induced Ab responses were assessed by conventional peptide ELISA and detection was performed using commercially available anti-mouse IgG1 and IgG2a second step reagents. Results showed that CCV-induced IgG are skewed in favor of IgG1, suggesting the development of a polarized humoral immune response towards the anti-inflammatory Th2 phenotype, consistent with existing literature. WISIT vaccines on the other hand produced a more balanced IgG1/IgG2 response, suggesting development of a more balanced Th1/Th2 immune response, respectively (Fig 7).

### WISIT vaccines suppress IL-31 signaling more effectively

Lastly, the ability of WISIT vaccine-induced Abs to suppress IL-31 signaling was assessed using A549 cells, a human epithelial carcinoma cell line expressing both hIL-31RA and OSMR, which are required for IL-31 signaling induction. In A549 cells, hIL-31 induces STAT3 phosphorylation and subsequent IL-31 signaling [34]. Results demonstrated that several of the tested peptides (Pep1, Pep2, Pep4, Pep9 and Pep10) were capable of inducing Abs inhibiting IL-31 signaling when presented by either WISIT vaccines or CCVs. However, WISIT vaccine-induced Abs targeting these peptides provided stronger inhibition of IL-31 signaling than the corresponding CCV-induced Abs for all inhibiting peptides (Table 2).

**Table 2:** Inhibition of STAT3 phosphorylation in A549 cells by Abs applied at 100ng/ml targeting the 10 peptides covering the IL-31 molecule, expressed as the mean % inhibition (n = 3).

Candidate	WISIT	CCV
Pep1	50%	30%
Pep2	53%	32%
Pep3	n.i.	n.i.
Pep4	75%	30%
Pep5	n.i.	n.i.
Pep6	21%	15%
Pep7	n.i.	n.i.
Pep8	n.i.	n.i.
Pep9	67%	35%
Pep10	60%	25%
Control	58%	

n.i… no inhibition.

**Fig 8:**
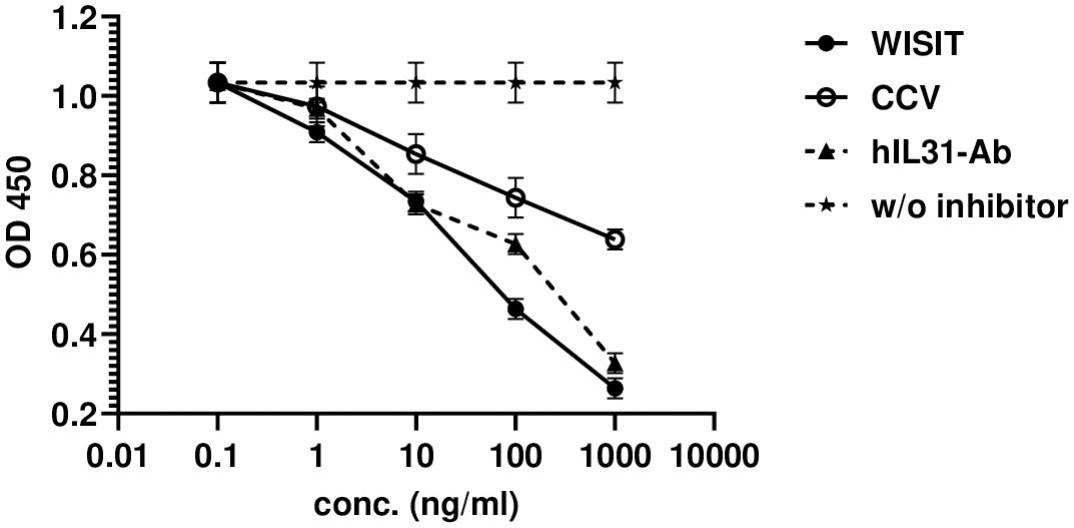
WISIT vaccine-induced Abs targeting Pep4 inhibit IL-31-induced STAT3 signaling more effectively than CCV-induced Abs in the A549 cell line (n = 3). Data is expressed as the mean. Error bars indicate the mean ± SEM.

Interestingly, at the concentration tested (100ng/ml Ab) CCV-induced Abs were unable to inhibit IL-31-mediated Stat-3 phosphorylation by more than 35%, whereas the corresponding WISIT vaccine-induced Abs were able to inhibit up to 75%. Pep4-, Pep9- and Pep10-WISIT vaccine-induced Abs even outperformed the commercial positive control anti-IL-31 Ab, achieving 58% inhibition in this assay. IL-31 signaling inhibition was also shown to be dose-dependent using Pep4-induced Abs as a representation (Fig 8).

However, not all IL-31 peptide candidate vaccines tested were capable of inducing Abs inhibiting IL-31 signaling. Pep3-, Pep5-, Pep7- and Pep8-induced Abs did not inhibit signaling when presented either by WISIT vaccines or CCVs (Table 2). For Pep5, Pep7 and Pep8, which induce strong anti-peptide Ab response, this is most probably due to the lack of induction of Abs that cross-react with full-length human IL-31 protein (Fig 3). Notably, Pep3-based vaccines induced strong anti-protein Ab responses with high avidity to IL-31 protein (Figs 4 and 6), when presented by WISIT vaccines, but Abs failed to inhibit IL-31 signaling in vitro. This finding underscores the importance of the selected epitope region for the inhibitory efficacy of the vaccines tested: only those regions that allow interference with Ab-receptor interaction or receptor activation will also actively inhibit signaling induction.

The results also correspond with the significantly higher avidity of the Ab responses generated for IL-31-candidate peptides when presented by WISIT vaccines compared to CCVs (Fig 6). A correlation analysis focused on three vaccines (i.e., Pep1, Pep2 and Pep4) all capable of inhibiting IL-31 signaling by targeting the correct epitopes, showed that Ab avidity for the IL-31 protein strongly correlated with the ability to inhibit IL-31 signaling in vitro (Pearson R^2^ = 0.7897). In contrast, CCV-type vaccine-induced Abs showed only a weak positive correlation (Pearson R^2^ = 0.2239). This analysis further supports the functional relevance of features such as Ab avidity for the target structures mediated by WISIT vaccines.

## Discussion

The results provided here demonstrate that WISIT vaccines are a promising future treatment option for CP, providing preclinical evidence of the potential benefits of WISIT technology for targeting IL-31 and identifying several candidate IL-31 peptide sequences to be pursued in future studies. The results show that, compared to CCVs, WISIT vaccines generate higher titers of anti-hIL-31 Abs with higher avidity to IL-31 and better-balanced IgG1/IgG2 ratios, which results in more effective IL-31 signaling suppression. WISIT vaccines targeting peptides Pep4, Pep9 and Pep10 performed most effectively in this work, and so these constructs will be the preferential candidates to be taken forward for further preclinical/clinical development.

As mentioned previously, immunogenicity is a combined metric dependent on both Ab concentration and target avidity, and avidity often correlates with the level of protection conferred by a treatment, which has been demonstrated across various vaccines and mAb treatments [35–37]. High titers of Ab with low avidity, or low titers with high avidity, do not elicit complete vaccine protection [32]. Indeed, vaccines generating low avidity Abs can even be detrimental, enhancing the pathology of the diseases being treated [38].

WISIT vaccines however appear to consistently produce anti-IL-31 Abs at high titers with high avidity, which is indicative that the resulting Ab response is likely to be highly effective against CP. However, as demonstrated in this report, high titers and avidity alone do not necessarily equate to an efficacious immune response.

IL-31 peptide Pep3 for example induced high Ab titers against IL-31 with high avidity but did not inhibit IL-31 signaling. A likely reason for this is that such non-functional Abs bind to the wrong regions, and so do not interfere with receptor binding/ligand activation [39,40]. For example, Pep1, Pep2 and Pep3 are located in close proximity, within 35 aas of Helix A, in IL-31. Although all three can mount high anti-protein titers only Pep1 and Pep2 mount a bioactive Ab response whereas Pep3-induced Abs fail to inhibit IL-31 signaling in vitro. Given their relatively modest superiority when formulated as WISIT vaccines compared to CCV, Pep1 and Pep2 will not be pursued in first instance. Several candidate-peptides targeting Helices B and Helix C/D used in this screen were either inducing Abs with low/absent cross-reactivity to IL-31 protein (Pep5, Pep7 and Pep8) and hence also failed in subsequent inhibition studies whereas vaccines targeting closely located epitopes (e.g.: Pep4, Pep9 and Pep10) induce Abs, which bind to IL-31 very efficiently and interfere with downstream IL-31 signaling. So, despite promising initial immunogenicity results for many WISIT candidates, careful target peptide selection is imperative.

The efficacy of WISIT vaccines, based on inhibition of in vitro IL-31 signaling, was found to depend on two main features. The location of the B cell epitope and the avidity of the Abs induced towards hIL-31. With respect to the latter, WISIT vaccines are characterized by a dramatically improved avidity for the IL-31 protein, the pathological component of CP, compared to the corresponding CCV vaccines. The functional relevance of the high antibody avidity is supported by our finding of a significant and direct correlation between antibody avidity and the ability to inhibit IL-31-mediated Stat3 phosphorylation in vitro. Given that WISIT vaccines also induce higher Ab titers compared to corresponding CCVs, it is reasonable to expect that WISIT vaccines will outperform CCVs in proof-of-concept type efficacy studies in vivo. Application of WISIT vaccines into the dermis layer showed to be effective in generating higher Ab responses when compared to s.c. and i.m. methods. While less commonly applied in the clinical setting, advancements in i.d. administration methods such as devices used to position vaccines into the dermis could help scale this method of administration in humans.

Furthermore, the demonstration that the WISIT vaccine-induced Abs are balanced between Th1 and Th2 responses could also show promise. A balanced Th1 and Th2 response is beneficial to the induction of an effective immune response [41]. Whilst Th2 responses are central to humoral immunity, excessive type 2 immune responses are a disease hallmark for dermatological disorders, promoting IgE and eosinophilic responses [42]. CCVs frequently induce Th2-biased responses, which are often associated with the presence of adjuvants such as alum [43–46], which may thus exacerbate CP. The induction of a balanced Th1/Th2 response in treating CP is not yet known, but WISIT vaccines show promise in inducing a more effective antibody response, creating a new paradigm of treating CP. Preclinical limitations resulted in testing of WISIT vaccines only in wt mice, accompanied by in vitro assessment of functional inhibition of human IL-31-signaling in vitro. Future research efforts will focus further on in vivo validation in additional animal models such as cynomolgus monkeys, dogs, and horses who are able to exhibit itching as a response to IL-31 overexpression and or administration. Furthermore, testing in humanized animal models will help bridge the gap between the preclinical and in human nature of these investigations.

## Conclusion

This report builds upon WISIT’s previous preclinical success targeting aSyn in Parkinson’s Disease 27] which is due to begin phase I clinical study in early 2025 [47], demonstrating that the WISIT platform is not disease-specific, but has the potential for application to a wide variety of diseases, including pruritic dermatological disorders driven by IL-31. The data presented in this study demonstrate the IL-31 WISIT vaccine technology (B-WISIT) to be superior to CCVs regarding both the titer of Abs induced to IL-31 B cell epitopes and the avidity of the Abs elicited. This translates into a functional benefit since Abs induced by WISIT vaccines inhibit human IL-31 signaling in vitro more efficiently than Abs triggered by their CCV counterparts. It will be necessary to follow up this work with studies investigating the in vivo efficacy of WISIT vaccines in appropriate animal models, as well as GLP-type safety and tolerability studies to optimize for the eventual testing of WISIT IL-31 vaccine candidates in humans.

## Supporting information

S1 Dataset (PDF)
