# Supplementary material for: WISIT vaccines based on IL-31-derived peptides as a novel therapeutic approach for chronic pruritic dermatoses
Source: PLoS One. 2025 Feb 11;20(2):e0318293. doi: 10.1371/journal.pone.0318293 (PMC11813111; doi:10.1371/journal.pone.0318293)
Supplement: S1 Dataset — (PDF) [file pone.0318293.s001.pdf]

Fig #	Mean	SD	SEM	Statistic Method used	P value	# samples
**Fig. 2a**						
WISIT	3,897	1,211	0,856			1 (triplicate)
CLEC	0,4405	0,06017	0,04255			1 (triplicate)
CCV	unstable					1 (triplicate)
**Fig. 2b**						
WISIT	1,737	0,03557	0,02054			1 (triplicate)
CLEC	0,08897	0,005201	0,003003			1 (triplicate)
CCV	unstable					1 (triplicate)
**Fig.2c**						
Pep1	9,659	0,4441	0,2564			1 (triplicate)
Pep3	4,494	0,3245	0,1874			1 (triplicate)
Pep4	1,737	0,03557	0,02054			1 (triplicate)
Pep5	16,13	1,943	1,122			1 (triplicate)
Pep6	9,326	0,244	0,1408			1 (triplicate)
Pep7	3,294	0,3644	0,2104			1 (triplicate)
Pep8	1,804	0,02307	0,01332			1 (triplicate)
Pep9	9,865	0,3755	0,2655			1 (triplicate)
Pep10	1,749	0,04504	0,02601			1 (triplicate)
**Fig.3**						
**WISIT**						
Pep1	206760	96770	43277	One way ANOVA/Tukey's multiple comparisons test	* p < 0.05; ** p < 0.01	10
Pep2	244841	127903	57200			10
Pep3	143260	74414	33279			10
Pep4	146135	71996	32198			10
Pep5	91466	42410	18966			10
Pep6	212097	121126	54169			10
Pep7	267083	152956	68404			10
Pep8	819247	873709	390734			10
Pep9	2789621	2858331	1429166			10
Pep10	219372	72562	32451			10
**CCV**						
Pep1	198135	104730	46837			10
Pep2	121166	49669	22213			10
Pep3	91883	46026	20584			10
Pep4	30208	12723	5690			10
Pep5	4187	1861	832,4			10
Pep6	3989	1524	681,7			10
Pep7	41580	16345	7310			10
Pep8	252514	144365	64562			10
Pep9	792060	772017	345257			10
Pep10	182396	58909	26345			10
**Fig.4**						
**WISIT**						
Pep1	18,54	7,322	4,227			10
Pep2	22,78	4,605	2,659			10

Pep3	36,88	16,47	9,508	One way ANOVA/Tukey's multiple comparisons test	* p < 0.05; ** p < 0.01; ***p<0.001	10
Pep4	15	2,069	1,195			10
Pep5	b.d.					10
Pep6	18,48	5,605	3,236			10
Pep7	b.d.					10
Pep8	b.d.					10
Pep9	7,243	2,027	1,17			10
Pep10	42,15	5,497	3,174			10
**CCV**						
Pep1	5,213	1,637	0,9448			10
Pep2	2,604	0,8837	0,5102			10
Pep3	1,985	0,6061	0,3499			10
Pep4	2,999	1,146	0,8106			10
Pep5	b.d.					10
Pep6	2,215	0,5561	0,3211			10
Pep7	b.d.					10
Pep8	b.d.					10
Pep9	4,661	0,9836	0,5679			10
Pep10	8,346	2,676	1,545			10
**Fig.5**						
**WISIT**						
i.d.	213099	120215	53762	One way ANOVA/Tukey's multiple comparisons test	** p < 0.01; ***p<0.001; ****p<0.0001	6
s.c.	71247	14631	6543			6
i.m.	4124	1849	924,5			6
**CCV**						
i.d.	25122	10378	4641			6
s.c.	75247	12911	5774			6
i.m.	44154	22611	10112			6
**Fig.6**						
**WISIT**						
Pep1	5,178	1,731	0,7743	One way ANOVA/Tukey's multiple comparisons test	* p < 0.05; ** p < 0.01	5
Pep2	3,778	1,291	0,5773			5
Pep3	8,846	2,911	1,456			5
Pep4	11,93	4,296	2,148			5
**CCV**						
Pep1	2,12	0,9654	0,4317			5
Pep2	2,08	0,7338	0,3282			5
Pep3	5,746	1,668	0,8341			5
Pep4	3,423	1,048	0,4687			5
**Fig.7**						
WISIT	153,3	25,17	14,53			5
CCV	2424	251,7	145,3			5
**Table 2**						
**WISIT**						
Pep1	50,52	1,54	1,089			3
Pep2	53,02	5,075	3,589			3
Pep3	97,47	0,8348	0,482			3

Pep4	75,26	1,039	0,7347			3
Pep5	97,57	1,106	0,6386			3
Pep6	21,57	0,6302	0,4456			3
Pep7	97,7	0,9	0,5196			3
Pep8	95,7	1,054	0,6083			3
Pep9	66,88	0,7792	0,551			3
Pep10	61,17	7,293	5,157			3
**CCV**						
Pep1	30	0,6562	0,464			3
Pep2	32,57	0,8838	0,6249			3
Pep3	97,57	1,106	0,6386			3
Pep4	29,27	14,05	9,932			3
Pep5	97,2	1,1	0,6351			3
Pep6	15,04	0,2546	0,18			3
Pep7	97,87	0,5859	0,3383			3
Pep8	96,53	1,069	0,6173			3
Pep9	34,12	7,185	5,08			3
Pep10	26,03	0,7153	0,5058			3
**Fig.8**						
**WISIT**						
0	1,069	0,03536	0,01768			3
1	0,9336	0,03536	0,01768			3
10	0,76	0,03536	0,01768			3
100	0,486	0,03329	0,01665			3
1000	0,286	0,03329	0,01665			3
**CCV**						
0	1,084	0,5759	0,288			3
1	0,9985	0,03536	0,01768			3
10	0,8785	0,03536	0,01768			3
100	0,766	0,03329	0,01665			3
1000	0,6616	0,03329	0,01665			3
**hIL31 Ab**						
0	1,07	0,03536	0,01768			3
1	0,993	0,03536	0,01768			3
10	0,7521	0,03536	0,01768			3
100	0,6496	0,03329	0,01665			3
1000	0,3496	0,03329	0,01665			3
**control**	1,077	0,006481	0,00324			3
